# Intra-Recto-Abdominal Exploration

**Published:** 1873-12

**Authors:** 


### Intra-Recto-Abdominal Exploration.

From proceedings N. Y. Academy of Medicine Record, Dec. 1st.

The first paper for the evening was read by Dr. Charles A. Leale, entitled "Intra-recto-abdominal Manual Exploration."

The speaker first called attention to the important assistance which this means of examination may give in diagnosticating various conditions of the viscera found in the lower portion of the abdominal cavity, such as are immediately connected with the bladders, uterus, ovaries, and even as high up as the kidneys, stomach and liver, and spleen. The method of procedure was described as follows: The patient upon whom the operation was performed was already perfectly relaxed, having swallowed a poisonous dose of chloroform. The hand and arm were well smeared with some lubricating substance, the hand, closely folded in a cone shape, was carefully passed through the sphincter ani, and then a moment's pause was made; the hand was carried on until it reached the sigmoid flexure of the colon, when it was partially turned upon itself, permitting the back of the hand to look towards the concavity of the flexure, and then permitted to pass through the flexure, when another pause was made; from this point the hand was carried along the colon until it reached a point four inches above the umbilicus, then being sixteen inches from the anus, as measured upon the arm. While in this position the fingers could be distinctly felt through the abdominal walls, and were readily recognized by an assistant. As the hand was being withdrawn, an exploration was made of some of the pelvic organs, although this was not the object which gave rise to the introduction of the hand, as will be seen further along. The patient was a sufferer from dysmenorrhœa. The uterus was found displaced, and could be as easily grasped and examined as if being examined through an opening made in the walls of the abdomen. An interstitial fibroid was also recognized upon its surface. The ovaries were grasped and the bladder recognized. The hand was then withdrawn without being stained in the least by blood. The greatest dilatation of the anus was ten inches in circumference, corresponding to the size of the forearm.

The operation was performed substantially as described by Prof. Simon, of Heidelberg, to whom is due the great advance which this method of exploration has made within a few years past. A brief history of the case in which the operation was performed by Dr. Leale, and the reason for performing it, is as follows:

Mrs. H., æt. 45, drank two ounces of pure chloroform for a suicidal purpose. As soon as the Dr. arrived, an emetic of salt and water was immediately administered, and which almost as immediately produced free emesis. No odor of chloroform was detected from the contents of the stomach. Strong odor of chloroform was recognized in the breath, showing that the chloroform had been entirely absorbed, and was being eliminated rapidly by the lungs. The stomach was washed out in addition to the emesis. The patient remaining insensible, and giving unmistakable evidences that death from asthenia was rapidly approaching, it was determined to pass the hand into the abdominal cavity, and irritate the solar plexus of nerves with the hope that respiration might be sufficiently aroused to carry the woman over the extreme point of danger. The result of the operation certainly justified its performance; for simultaneously with the irritation of the root of the diaphragmatic plexus of nerves the respiratory act became more vigorous, was soon followed by returning consciousness, and the patient was saved from a fatal narcotism.

Three days after the operation the woman was again at work, the sphincter retained the fæces in a normal manner, there was no gastritis, and recovery was made without an unpleasant symptom, save a sharp headache which lasted a few days.

Irritation of the solar plexus of nerves in this case was suggested by the results obtained, in a case of like character, from the use of electricity over the region of the solar plexus, for the purpose of increasing the force of the respiration. In that instance the patient was restored from impending death by the use of artificial respiration, and the application of electricity as referred to.

Dr. Benjamin Howard commented upon Dr. Leale's paper substantially as follows: I feel hardly able to share the enthusiasm expressed by the author of the paper respecting the employment of internal abdominal explorations in the diagnosis of pathological conditions within that cavity. I am of the opinion that there is a debit and credit side to the question.

From this point the doctor very pleasantly illustrated his ideas by drawings upon the blackboard.

In the first place, with regard to its value as a means of resuscitation. Although Dr. Leale has fortified his case by the results obtained, I am not sure that the same results might have been obtained by striking sharply upon the epigastrium, where we can come into very close proximity with the solar plexus, and produce marked effect by blows. I am of the opinion that, although admissable, the use of both electrity and internal abdominal exploration, for the purpose of irritating the solar plexus of nerves as a means of resuscitation, should only be employed as a *dernier ressort*, and for the reason that less formidable and equally efficient means can be obtained by externally slapping the epigastric region.

The Doctor mentioned some precautions to be taken in the performance of the operation, to which the author of the paper did not make special reference. The use of anæsthetic is always required to render the operation possible. The anus should be fully dilated, by making firm traction with the fingers inserted into it, and making traction in opposite directions. As soon as the promontory of the sacrum is reached, the hand should be gently turned, bringing the back of the hand uppermost, to facilitate its passage through the sigmoid flexure.

With regard to the value of this method as a means of diagnosis of stone in the bladder. When the bladder is full, a calculus lying in the *trigone vesicæ* may be raised upon the ends of the fingers, and it would seem as if it could be grasped in the hand, but that cannot be done. The space is so small, and so much tissue intervenes between the hand and the stone, that it is impossible to seize it. Some idea of the weight of the stone may be derived by this method, and that is all.

I am of the opinion that this method of examination promises to be an exceedingly valuable one in the diagnosis of tumors of the uterus, ovarian tumors, displacement of the uterus, etc. By this means much can be learned of the density and connections of tumors existing in the pelvic cavity. That which would most justify this method of examination, is the existence of a stricture, especially when it occurs at the sigmoid flexure of the colon. The stricture can be much more advantageously reached with the fingers than with a bougie, and some estimation can be made of its character and extent, which can scarcely be derived by any other means than the touch.

So much for the advantages attending its use. There is, however, a debit side of the question. In some cases, where the hand has been introduced with apparent success, and the amount of dilatation has been very moderate, a good deal of difficulty in controlling the fæces has followed. In those cases where it is most justifiable it may do the most harm, perhaps. There is great liability, in case of stricture of the intestine, of having ulcers just before or just beyond the stricture, and the violence necessary for the introduction of the hand into the bowel may be sufficient to convert the ulceration into a perforation, and with this comes collapse and death. Such cases have already been recorded in *The Lancet*, and should have an influence upon the employment of the method of examination. Too much should not be expected from its adoptioner and it should be resorted to only in exceptional cases, and perhaps only as a *dernier ressort*.

Dr. Peaslee would place a check upon the indiscriminate use of this method of exploration. It can be employed with benefit in certain cases, in which abortion has habitually occurred in consequence of displacement of the uterus backward. If resorted to for this purpose it is probably well to wait until the pregnancy has advanced to four months, and it should not be longer postponed for reasons which are evident. If practiced earlier than this, the uterus will immediately fall back, and no benefit will be derived.

The use of this method in gynæcology is very important, and so long as the hand is kept within the pelvis, no harm can well be done; but as soon as the hand passes above, there is a liability to do injury which corresponds to the size of the hand and the size of the intestine.

In some cases the curves of the intestine are such that it is impossible to pass beyond them without lacerating the gut. Its chief use should be restricted to exploration in connection with diseases within the pelvis The bowels should be thoroughly emptied before the operation; and when highly exploration is resorted to, throwing a stream of warm water alongside of the arm, by means of a long-nozzled syringe, will materially facilitate its passage. The operation, in general, should be employed only in exceptional cases.

It is justifiable when a differential diagnosis is to be made between a fibroid cyst of the uterus and an ovarian tumor, or whether the tumor is an ovarian tumor or a tumor which comes from above, such as an enlarged kidney or liver. Still it should not be resorted to, only when other means have failed in arriving at a diagnosis. As a rule, the loss of power in controlling the passage of the fæces lasts only for a few days; yet this is one reason against an indiscriminate use of the method.

Dr. Leale was of the opinion that, with a very pendulous abdomen, such as his patient had, irritation of the solar plexus could not be as satisfactorily accomplished by external measures as by internal.

